# The HepTestContest: a global innovation contest to identify approaches to hepatitis B and C testing

**DOI:** 10.1186/s12879-017-2771-4

**Published:** 2017-11-01

**Authors:** Joseph D. Tucker, Kathrine Meyers, John Best, Karyn Kaplan, Razia Pendse, Kevin A. Fenton, Isabelle Andrieux-Meyer, Carmen Figueroa, Pedro Goicochea, Charles Gore, Azumi Ishizaki, Giten Khwairakpam, Veronica Miller, Antons Mozalevskis, Michael Ninburg, Ponsiano Ocama, Rosanna Peeling, Nick Walsh, Massimo G. Colombo, Philippa Easterbrook

**Affiliations:** 1 University of North Carolina Chapel Hill Project-China, Number 2 Lujing Road, Guangzhou, 510095 China; 2 Institute of Global Health and Infectious Diseases, University of North Carolina Chapel Hill, 130 Mason Farm Rd, CB# 7030, Chapel Hill, NC 27599-7030 USA; 3 SESH Global, Number 2 Lujing Road, Guangzhou, 510095 China; 4 Aaron Diamond AIDS Research Center, 455 1st Avenue # 7, New York, NY 10016 USA; 5 University of Pennsylvania Neurology Department, 3400 Spruce Street, Philadelphia, PA 1914 USA; 6 Asia Catalyst, 1109, 1270 Broadway, New York, NY 1001 USA; 7 WHO Regional Office for South East Asia, World Health House, Indraprastha Estate, Mahatma Gandhi Marg, New Delhi, Delhi 110002 India; 8 Southwark Council, 160 Tooley Street, London, SE1 2QH UK; 9 Médecins Sans Frontières, Rue de Lausanne 78, 1202 Genève, Switzerland; 10 World Health Organization HIV Department, 20 Avenue Appia, CH-1211 Geneva 27, Switzerland; 11 Forum for Collaborative HIV Research and the University of California Berkeley School of Public Health, 1608 Rhode Island Avenue NW, Suite 212, Washington, DC 20036 USA; 12 World Hepatitis Alliance, 1 Baden Place, London, SE1 1YW UK; 13 Hepatitis C Trust, 27 Crosby Road, London, SE1 3YD UK; 14 TREAT Asia, Exchange Tower, 388 Sukhumvit Road, Suite 2104, Klongtoey, Bangkok 10110 Thailand; 15 WHO Regional Office for Europe, UN City, Marmorvej 51, DK-2100 Copenhagen, Denmark; 16 Hepatitis Education Project, 1621 S. Jackson Street, 2t 201, Seattle, WA 98144 USA; 17 Department of Medicine, Makere College of Health Sciences, PO Box 7072, Kampala, Uganda; 18 London School of Hygiene and Tropical Medicine, Keppel Street, London, WC1E 7HT UK; 19 The WHO Regional Office for the Western Pacific, P.O. Box 2932, 1000 Manila, Philippines; 20 IRCCS Humanitas Hospital, Rozzano, Italy; 21 EASL International Liver Foundation, Geneva, Switzerland

## Abstract

**Background:**

Innovation contests are a novel approach to elicit good ideas and innovative practices in various areas of public health. There remains limited published literature on approaches to deliver hepatitis testing. The purpose of this innovation contest was to identify examples of different hepatitis B and C approaches to support countries in their scale-up of hepatitis testing and to supplement development of formal recommendations on service delivery in the 2017 World Health Organization hepatitis B and C testing guidelines.

**Methods:**

This contest involved four steps: 1) establishment of a multisectoral steering committee to coordinate a call for contest entries; 2) dissemination of the call for entries through diverse media (Facebook, Twitter, YouTube, email listservs, academic journals); 3) independent ranking of submissions by a panel of judges according to pre-specified criteria (clarity of testing model, innovation, effectiveness, next steps) using a 1-10 scale; 4) recognition of highly ranked entries through presentation at international conferences, commendation certificate, and inclusion as a case study in the WHO 2017 testing guidelines.

**Results:**

The innovation contest received 64 entries from 27 countries and took a total of 4 months to complete. Sixteen entries were directly included in the WHO testing guidelines. The entries covered testing in different populations, including primary care patients (*n* = 5), people who inject drugs (PWID) (*n* = 4), pregnant women (*n* = 4), general populations (*n* = 4), high-risk groups (*n* = 3), relatives of people living with hepatitis B and C (*n* = 2), migrants (*n* = 2), incarcerated individuals (*n* = 2), workers (*n* = 2), and emergency department patients (*n* = 2). A variety of different testing delivery approaches were employed, including integrated HIV-hepatitis testing (*n* = 12); integrated testing with harm reduction and addiction services (*n* = 9); use of electronic medical records to support targeted testing (*n* = 8); decentralization (*n* = 8); and task shifting (*n* = 7).

**Conclusion:**

The global innovation contest identified a range of local hepatitis testing approaches that can be used to inform the development of testing strategies in different settings and populations. Further implementation and evaluation of different testing approaches is needed.

**Electronic supplementary material:**

The online version of this article (10.1186/s12879-017-2771-4) contains supplementary material, which is available to authorized users.

## Background

Hepatitis B virus (HBV) and HCV infection are major causes of chronic liver disease globally and together account for about for about 1.34 million deaths per year, mainly from cirrhosis or hepatocellular carcinoma [1, 2]. The recent development of highly effective, well-tolerated oral treatment regimens with high rates of cure after 12 weeks of treatment has revolutionized the treatment of chronic HCV infection [3]. Effective long-term antiviral treatment with tenofovir or entecavir is also available for people with chronic HBV infection [4, 5]. Despite the high burden of disease and treatment advances, the majority of people infected with HBV [6, 7] and HCV [8–10] remain unaware of their infection. Estimates from the 2017 Global Hepatitis Report indicated that only 9% of persons living with HBV (22 million) and 20% of persons living with HCV (14 million) had been tested and were aware of their diagnosis [2]. There are several potential reasons for insufficient hepatitis testing, including limited facilities for hepatitis testing, lack of national testing policies, costly diagnostic assays, complicated diagnostic algorithms, and inadequate quality assurance systems. A further barrier has been the lack of service delivery models for hepatitis testing in different settings and populations.

There are many potential facility or community based approaches to delivery hepatitis testing [11]. Facility-based opportunities include primary care and outpatient clinics such as specialist clinics (e.g., HIV, TB and STI clinics), inpatient services, and private services. Community based testing can be offered through outreach and mobile programmes in schools and workplaces. Although many of these approaches were developed to expand HIV testing [12], some of these may also be relevant to expand viral hepatitis testing. WHO has recently developed evidence-based testing guidelines for hepatitis B and C virus infection [13]. The evidence-base included several systematic reviews but recommendations were also based on consideration of overall benefits and harms, cost-effectiveness, resource use, acceptability, and programmatic feasibility using the GRADE criteria [14]. However, in contrast to HIV care for which there is an extensive literature on different options for HIV testing through health facilities and in different populations, there is limited published literature on programmatic experience of hepatitis testing [11, 13]. To address the limited published literature and evidence-base to support different service delivery approaches to hepatitis testing in the World Health Organization Hepatitis Testing Guidelines, we used an innovation contest to solicit a range of approaches to hepatitis B and C testing. Innovation contests allow communities to identify novel approaches to implementing public health interventions, often with the help of the internet and multisectoral partnerships [15]. The goal of the contest was to enrich the guidelines with real-world practices in different settings and populations. Previous health-focused innovation contests solicited public health concepts, images [16, 17], and videos [18, 19] to promote public health campaigns in support of HIV testing [16, 19], healthy sexual behavior [16, 20, 21], healthy eating habits [22–24], regular sunscreen use [25, 26] and smoking cessation [27–29].

Common themes of these contests are the issuing of an open call for entries, engaging a range of individuals and groups, evaluation of entries, and recognition of finalists [16, 30]. The purpose of this paper is to describe the methodology of an innovation contest and key findings from the range of submissions.

## Methods

### Innovation contests

The innovation contest was organized through a collaboration between SESH (Social Entrepreneurship for Sexual Health) and the World Health Organization Global Hepatitis Programme. SESH has organized a total of 16 innovation contests [16, 18, 30]. The contest was initiated on December 15th 2015 with the formation of a steering committee and was completed on April 17th 2016 with the announcement of finalists and issuing of commendation certificates at the 2016 European Association for the Study of the Liver (EASL) conference. The contest had several discrete steps: 1) establishment of a multisectoral steering committee to plan the contest; 2) dissemination of the call for entries through diverse media; 3) independent evaluation and ranking of entries by a panel of judges according to pre-specified criteria; 4) recognition of contestants through commendation and case studies in the WHO testing guidelines.

### Establishment of a multisectoral steering committee to plan the contest

A multisectoral steering committee was established with representation from key stakeholders and geographic regions. These included WHO (HQ Global Hepatitis Programme and regional office focal points in hepatitis), public health experts, implementing partners, social media and communications experts, and advocacy organisations. The steering committee guided the overall development, evaluation, and promotion of the contest coordinated through a series of teleconferences. An open call was made January 15th 2016 that explained the purpose of the contest and solicited short descriptions of programmes to promote hepatitis B and C testing. Entries were to include a description of the testing model; a description of how it is novel with regard to promoting uptake of testing in either setting, population or education; data to support evidence of effectiveness and impact of programme, including linkage to care; and next steps for continuing the work and ensuring sustainability (Additional file 1). Eligible entries were those that described existing ongoing programmes. The announcement was translated into the six UN languages (French, Chinese, Arabic, Russian, Spanish, English) and included a link to a one-minute video explaining the contest, its purpose, and how people could contribute (Additional file 1). The hashtag “HepTestContest” identified the campaign on social media platforms. All contestants were also asked to complete a brief online survey that asked when their testing programme started, what proportion of the organization’s work was focused on hepatitis, the availability of direct acting antivirals in their country, whether or not they also provided HIV testing, and perceived barriers to testing.

### Dissemination of the call for entries through diverse media

In order to disseminate the call for entries widely, we used several social media platforms (Twitter, Facebook, and YouTube), as well as the email listservs and networks of collaborating organisations represented by the steering committee. The call for entries was also included in two academic journals, *Journal of Virus Eradication* and *Hepatitis, Policy, and Medicine*. The hashtag “HepTestContest” was used to identify the campaign on social media platforms. We used real-time social media analytics from a video hosting site, innovation contest webpage, Twitter hashtag activity, and submissions to assess both total and regional engagement during the 6 weeks of soliciting entries.

### Independent evaluation of entries by a panel of judges according to pre-specified criteria

In addition to the Steering committee members, additional persons were invited to participate in rating the entries to increase representation from under-represented regions and community-based organizations (CBOs). A total of sixteen judges participated. Each judge independently rated each of the four parts of the abstract submission on a 1-10 scale: description of testing model, innovation, effectiveness, and next steps for sustainability, with 10 indicating a high and 1 a low score. In addition, each judge gave an overall score of 1-10. Judges who had a conflict of interest with a programme recused themselves from reviewing that entry. The three non-English entries were evaluated by judges proficient in the language of the entry. Conflicts were defined as collaborating, co-authoring, helping, receiving or providing monetary or other support, or anything that could be perceived as a conflict of interest. Judges entered their scores into an online scorecard developed in SurveyGizmo (Boulder, USA), a software programme for creating online surveys. The software facilitated data analysis and provided a common platform to securely enter scores. An overall mean score that averaged the means for each of the four subcomponents (testing model, innovation, effectiveness, next steps) was calculated.

### Recognition of a subset of contestants through commendation and inclusion in the WHO testing guidelines

Entries that received an overall mean score of greater than 6.0 (a threshold determined by the Steering Committee, representing 15% of all submissions) on the 1-10 scale received a commendation of excellence from EASL, on behalf of the Innovation Contest Steering Committee, SESH and WHO. In addition, five of the finalists were invited to present their testing innovations at the 2016 International Liver Congress (ILC)/EASL in Barcelona, Spain. One finalist also presented at the International AIDS Society conference (21 July 2016) in Durban, South Arica and at the World Hepatitis Day (28 July 2016) in Mumbai, India. In addition, selected submissions were included as case studies in the chapter on service delivery within the WHO hepatitis testing guidelines.

## Results

### Key characteristics of contest entries

**Fig. 1:**
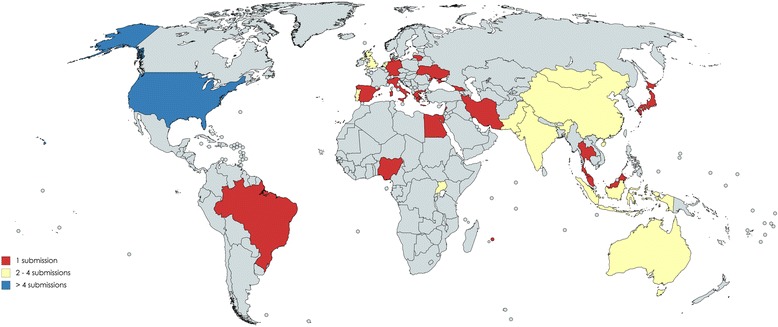
Map of countries with contributions to the hepatitis testing innovation contest

We received 64 entries from 27 countries in all WHO regions (Fig. 1). Among the 31 commended entries, these included two from Africa (one from Egypt, one from Nigeria), nine from Asia (three from India, two from Australia, one from Malaysia, one from Indonesia, one from Mongolia, one from China), six from Europe (three from the UK, one from Georgia, one from the Netherlands), and 14 from North America (all 14 from the US). Among commended entries, 12/31 (39%) were from non-government organizations, 10/31 (33%) were from hospitals, 6/31 (19%) were from research organizations, and 4/31 (13%) were from governmental organizations. Nearly all (61/64) entries were in English and we received one entry in Portuguese, one in Russian, and one in Chinese. Of the 64 entries, 21 (33%) tested for hepatitis B and C, 25 (39%) for hepatitis C only, and 10 (16%) for hepatitis B only. A total of 10/64 (16%) only tested for hepatitis B, 25/64 (39%) only tested for hepatitis C, and 21/64 (33%) tested for both. The median number of persons tested through each entry was 1367 (range 90 – 240,188) among the 39 entries that reported testing numbers. A majority (42/64, 66%) of entries were from testing programmes initiated in the past 3 years and most (45/64, 71%) programmes also provided HIV testing.

The 64 entries demonstrated a broad range of testing locations, populations, and service models (Table 1). In terms of testing locations, 18 programmes were based at clinical facilities; 11 were at non-clinical sites; two tested at courthouses or prisons; and one in the workplace. Populations tested in different programmes included six testing people who inject drugs (PWID); four in pregnant women; three among the general population; three in high-risk groups; two among relatives of people living with hepatitis B and C; two among migrants; two in incarcerated individuals. Eight used electronic medical records in primary care to flag higher-risk populations for testing. Five used social media to promote uptake of testing. In terms of service models, several programmes employed well–established health system programming practices to support testing scale-up, including delivery of integrated testing (12 HIV-hepatitis, and 9 with harm reduction or addiction services); task sharing (*n* = 8) and decentralisation (*n* = 8); and use of task shifting of testing activities to other cadres of health workers (*n* = 8).

**Table 1:** Commended entries from the global hepatitis testing innovation contest, 2016, organized by region (*n* = 31)

Continent	Country	Organization (Organization Type)	HBV/HCV	Population Tested	Setting	Key feature of service delivery	Health system programme practices
Africa	Egypt	Association of Liver Patients Care (NGO) and the Egyptian Liver Hospital (Hospital)	HBV/HCV	General population	Community (non-clinical)	SMS promotion Community empowerment	Social marketing, decentralization
Africa	Nigeria	Federal Ministry of Health (Government)	HBV/HCV	General population	Community (non-clinical)	Social media	SMS promotion, public-private partnership, task shifting
Asia	India	Community Network for Empowerment (NGO)	HCV	PWID, PLHIV, outpatients	Community (clinical and non-clinical)	Community empowerment	Decentralization, task shifting, public-private partnership
Asia	Mongolia	Flagstaff International Relief Effort (NGO)	HBV/HCV	Health care workers, social workers, and family history of liver cancer	Primary care clinics	Integration with primary care	Decentralization, integration with HBV immunization
Asia	India	Institute of Liver and Biliary Sciences (Research)	HBV/HCV	First degree relatives of HBV+ patients; PWID	Health facility clinics	Family-focused test promotion	Integration with HBV immunization, task shifting
Asia	India	Medecins Sans Frontieres (NGO)	HCV	PLHIV	Health facility clinics	HIV-hepatitis integration	Task shifting
Asia	Indonesia	Persaudaraan Korban Napza Indonesia (NGO)	HCV	PWID	Urban sites	Peer-based	HIV-hepatitis integration, addiction service integration
Asia	Australia	St Vincent’s Hospital Melbourne and Justice Department, Victorian State Government (Hospital)	HCV	Prisoners on entry or transfer	Prison	Prison-based telemedicine	Integration with primary care and addiction services
Asia	Australia	The Kirby Institute, UNSW Australia (Research)	HCV	PWID	Community (clinic and non-clinical)	Social media	Social marketing, addiction service integration
Asia	Malaysia	University of Malaya-Center of Excellence for Research in AIDS (Research)	HCV	PWID	Methadone clinic	Methadone clinic-based services	Harm reduction/addiction service integration, peer-based
Asia	China	Yunnan AIDS Initiative (NGO)	HBV	Pregnant women and partners	Maternal and child health clinics	Prenatal service integration	HIV-hepatitis integration
Europe	UK	Barts Health NHS Trust (Hospital)	HBV/HCV	Emergency department patients	Emergency department	Social media	HIV-hepatitis integration, public-private partnership
Europe	UK	Chelsea and Westminster Hospital (Hospital)	HBV/HCV	Emergency department patients (HIV-negative)	Emergency department	EMR, simplified pathway for emergency department testing	HIV-hepatitis integration,
Europe	Portugal	IN-Mouraria (NGO)	HBV/HCV	PWID	Substance abuse programs	Comprehensive harm reduction services for PWID	HIV-hepatitis integration, addiction service integration, peer-based, task shifting
Europe	UK	James Cook University Hospital (Hospital)	HBV/HCV	General population	Health facility clinics	EMR	HIV-hepatitis integration
Europe	Georgia	Georgian Harm Reduction Network (NGO)	HCV	PWID	Outreach mobile sites	Methadone-based service delivery for PWID	Decentralization, public-private partnership, task shifting
Europe	Netherlands	Public Health Service of Amsterdam (Government)	HCV	“At Risk” based on questionnaire	Health facility clinics	Web-based risk stratification and referral	Use of social media to promote access
North America	US	Emory University/Grady Health System: Grady Liver Clinic (Research/Hospital)	HCV	Birth year (1945-1965)	Primary care clinics	EMR	Decentralization
North America	US	Hep Free Hawaii (NGO)	HBV/HCV	General population	Pharmacies	EMR	Use of social media to promote access, decentralization
North America	US	Horizon Health Center (Hospital)	HCV	New hospital and clinic patients/and untested for HIV in previous 12 months	Health facility clinics	EMR	HIV-hepatitis integration
North America	US	Imagine Hope (NGO)	HCV	PWID	Methadone clinics and substance abuse programmes	Addiction service integration	HIV-hepatitis integration, public-private partnership
North America	US	Kaiser Permanente Mid-Atlantic States (Hospital)	HCV	General population & birth year	Primary care clinics	EMR	HIV-hepatitis integration
North America	US	Memorial Hermann Healthcare System (Hospital)	HCV	Birth cohort (1945-1965)	Emergency department	EMR	HIV-hepatitis integration, public-private partnership
North America	US	National Nursing Centers Consortium (NGO)	HCV	Outpatients	Primary care clinics	EMR	HIV-hepatitis integration, task shifting
North America	US	Philadelphia Department of Public Health (Government)	HCV	PWID, including homeless and sex workers	Syringe exchange programmes	Addiction service integration	Task shifting, peer-based
North America	US	Project IMPACT (NGO)	HCV	General population	Courthouse lobby and other sites	Courthouse integration	HIV-hepatitis integration, harm reduction.addiction service integration
North America	US	St Joseph’s Medical Center (Hospital)	HBV/HCV	General population and those with risk factors	Health facility clinics	EMR	NA
North America	US	St. Lukes-CHI/Project ECHO (Hospital)	HCV	Birth cohort and among those receiving STD tests	Primary care clinics	Telemedicine	Decentralization
North America	US	C a Difference, Drexel University (Research)	HCV	Outpatients	Primary care clinics	Social media	HIV-hepatitis integration, decentralization
North America	US	Virginia Department of Health, Division of Disease Prevention (Government)	HCV	PWID	Methadone clinics, substance abuse programmes	Addiction service integration	Decentralization, task shifting
North America	US	Asian Liver Center at Stanford University (Research)	HBV	Employees	Workplace	Workplace	Public-private partnership

*SMS* short message system, *HBV* hepatitis B virus, *HCV* hepatitis C virus, *PWID* people who inject drugs, *PLHIV* people living with HIV, *STD* sexually transmitted disease, *EMR* electronic medical records (for risk stratification and clinician reminders)

### Survey responses about organizations

Respondents to an additional survey showed that the majority (42/64, 66%) of entries were from testing programmes initiated in the past 3 years. Many of these (45/64, 71%) also provided HIV testing. The programmes highlighted a number of barriers to effective testing and treatment in the brief survey, which were common across countries, testing models, and types of organization. The most frequent barriers were lack of hepatitis awareness among the public and subpopulations (25/64, 39%), lack of testing supplies (25/64, 39%), insufficient funding (10/64, 16%), lack of knowledge among healthcare providers and community outreach workers (10/64, 16%), insufficient linkage (9/64, 14%), lack of drug access (8/64, 13%), stigma (4/64, 6%), and local or national laws on illicit drugs (3/64, 5%). A high proportion of organizations highlighted the need for additional support in terms of resources (50/64, 78%), communications (48/64, 75%), and technical expertise (35/64, 55%).

### Quality of entries

**Fig. 2:**
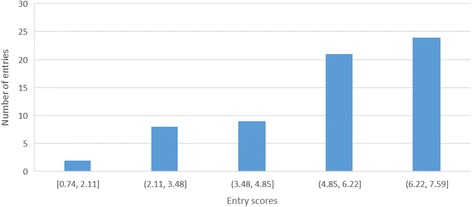
Histogram showing range of scores submitted to the global hepatitis innovation contest (*n* = 64)

In general, the quality of entries was high (Fig. 2), with an overall mean score across all judges and based on the four different component scores of 5.65. 31 (46%) of entries received an overall mean of greater than 6.0, and received a commendation certificate from EASL (Table 1). Sixteen submissions were included as short case studies in the chapter on service delivery model in the 2017 WHO hepatitis testing guidelines.

### Social media metrics

Video and website analytics suggested moderate engagement over the 6 weeks of the call for entries. By the end of the call for entries on March 1st, the video announcement had been viewed 453 times in 87 countries (this was 116 times in 37 countries within 2 weeks of the announcement). Website metrics showed that fewer individuals had accessed the announcements in Sub-Saharan African countries and in Latin America. For example, the respective number of website views early in the contest over 5 days (27-31 January) were: Africa (6), Americas (58), Eastern Mediterranean (2), Europe (114), South-East Asia (4), and Western Pacific (24). Access to both video and website analytics helped us to subsequently target further announcements through regional networks that reached those under-represented regions leading to modest increases in views from those regions. Targeting was achieved by the Steering Committee contacting regional networks to promote the contest within their specific region.

### Costs

We examined the costs of the contest solely from the perspective of the contest organisation, and not the costs associated with implementation of the testing approaches. There was no specific funding to support the conduct of the contest. All steering committee members and organizers of the contest volunteered their time, while ILC-EASL supported the cost of having finalists travel to and attend the EASL conference. The Office of the WHO in the Southeast Region supported the cost of travel and registration at the World Hepatitis Day event in India.

## Discussion

This global innovation contest led by a multi-sectoral steering committee attracted 64 entries describing different approaches to hepatitis B and C testing in 27 countries. Overall, the contest was straightforward to organize and implement over a four-month period. The contest elicited high-quality submissions from multiple institutions and community organizations across all geographic regions. There were several distinct positive outcomes from this initiative. First, the inclusion of a range of examples of different service delivery models for testing in different populations and settings as case studies in the 2017 WHO hepatitis testing guidelines will be of practical value to countries and programmes as they start hepatitis testing activities. Second, the contest succeeded in engaging multiple organisations who may be in a position to undertake more formal evaluation of their testing models, as well as provide input on future hepatitis testing and treatment policy. Third, the contest also served as an opportunity for non-governmental organizations to raise their profile and obtain recognition for their work.

The contest identified a range of hepatitis B and C testing approaches that can be used in both community settings and health facilities such as integrated HIV-hepatitis testing; use of social media to promote uptake of testing; workplace testing; testing camps; using emergency departments to promote testing; use of electronic medical record prompts to flag higher risk patients for targeted testing. In addition, the contest identified innovative testing approaches to reach different populations, such as PWID, prisoners and other high-risk groups, migrants and relatives of people living with hepatitis B or C.

The highlighting of the effectiveness of integration of hepatitis testing within other programmes (eg. HIV, mental health services), use of electronic and other reminders to prompt testing, and use of community-based lay providers to deliver testing is consistent with studies showing their effectiveness from both HIV [31] and hepatitis care [11]. In particular, the availability of an existing laboratory and clinical infrastructure, trained personnel and funding streams presents a key opportunity to build hepatitis testing into existing HIV testing programmes [32–35]. However, the development of integrated HIV and hepatitis testing will require additional training and resources [36].

The contest also identified a number of well recognized barriers to hepatitis testing, in particular poor awareness among the general public and limited access to test kits [37–39]. This finding is consistent with other reports from LMICs [37, 38]. Poor awareness of hepatitis B and C is likely related to many factors, including limited resources for campaigns [40], a relative lack of celebrity ambassadors for hepatitis [41, 42], and the asymptomatic nature of chronic viral hepatitis in many cases [43]. These challenges are consistent with experience reported from other innovation contests focused on promoting HIV testing [16, 19].

We identified several key features that may have contributed to success of this contest. First, the establishment of a diverse steering committee (including individuals in academia, civil society, government, WHO, medicine, and public health) helped disseminate the call for entries and evaluate entries. Second, we offered incentives to participate that included presentation at international conferences and potential inclusion within the WHO guidelines which may have been important in motivating participation. Although the profiles provided were brief, several contest submissions have since published more detailed descriptions of their programmes and evidence of impact [44–49]. Third, real-time tracking of social media analytics during the contest enhanced subsequent contest promotion towards under-represented regions.

However, there were several limitations to the use of a contest approach to identify innovative models. First, this contest relied on the internet, especially social media, to distribute the call for entries and submit an entry. As a result, contest entries were not representative of all regions, settings, testing models, and populations. We received fewer submissions from Latin America and Africa, and only three entries in non-English languages despite announcing the call for submissions in six languages. This may be related to insufficient dissemination through those regional networks, less internet access, and fewer ongoing hepatitis testing programmes in those settings. Other crowdsourcing contests have demonstrated the additional value of in-person promotion [16]. Second, the evaluation of quality of submissions was only based on a 300 word description, without further opportunity to clarify or extend. Importantly, only just over half of entries formally evaluated the impact of their programme, and we further noted that several programmes expressed challenges in funding, indicating uncertainty about the financial viability and sustainability of some of these approaches.

## Conclusions

In conclusion, our experience suggests that this contest was a useful way of eliciting a wide range of programmatic experience that can supplement and enrich formal evidence reviews to inform guidelines and policy, and maybe applicable to other areas. Case studies were included in the 2017 WHO viral hepatitis testing guidelines, as they were in the 2015 WHO consolidated guidelines on HIV testing [31]. The inclusion of good practice examples may serve as a useful model for future international guidelines. However, there remains a need for more systematic evaluation of these different service delivery approaches in different populations. Ultimately, countries will need to identify the most appropriate mix of facility and community based testing opportunities according to local epidemiology, current testing coverage, existing health-care and testing infrastructure and services, and available financial and human resources.

## Additional file

Additional file 1: HepTestContest Call for Entries. This provides the logo, call for entries, and related information released to solicit entries. (DOCX 38 kb)

## References

[1] Stanaway JD, Flaxman AD, Naghavi M, Fitzmaurice C, Vos T, Abubakar I, Abu-Raddad LJ, Assadi R, Bhala N, Cowie B (2016). The global burden of viral hepatitis from 1990 to 2013: findings from the global burden of disease study 2013. Lancet.

[2] World Health Organization (2017). Global hepatitis report.

[3] Gutierrez JA, Lawitz EJ, Poordad F (2015). Interferon-free, direct-acting antiviral therapy for chronic hepatitis C. J Viral Hepat.

[4] Bhattacharya D, Thio CL (2010). Review of hepatitis B therapeutics. Clin Infect Dis.

[5] Bitton Alaluf M, Shlomai A (2016). New therapies for chronic hepatitis B. Liver Int.

[6] van der Veen YJ, Voeten HA, de Zwart O, Richardus JH (2010). Awareness, knowledge and self-reported test rates regarding hepatitis B in Turkish-Dutch: a survey. BMC Public Health.

[7] Chung PW, Suen SH, Chan OK, Lao TH, Leung TY (2012). Awareness and knowledge of hepatitis B infection and prevention and the use of hepatitis B vaccination in the Hong Kong adult Chinese population. Chin Med J.

[8] World Health Organization (2016). Guidelines for the screening, care and treatment of persons with hepatitis C infection.

[9] Denniston MM, Klevens RM, McQuillan GM, Jiles RB (2012). Awareness of infection, knowledge of hepatitis C, and medical follow-up among individuals testing positive for hepatitis C: National Health and nutrition examination survey 2001-2008. Hepatology.

[10] Ng MH, Chou JY, Chang TJ, Lee PC, Shao WC, Lin TY, Chen VC, Gossop M (2013). High prevalence but low awareness of hepatitis C virus infection among heroin users who received methadone maintenance therapy in Taiwan. Addict Behav.

[11] Zhou K, Fitzpatrick T, Walsh N, Kim JY, Chou R, Lackey M, Scott J, Lo YR, Tucker JD (2016). Interventions to optimise the care continuum for chronic viral hepatitis: a systematic review and meta-analyses. Lancet Infect Dis.

[12] Chang TT, Liaw YF, Wu SS, Schiff E, Han KH, Lai CL, Safadi R, Lee SS, Halota W, Goodman Z (2010). Long-term entecavir therapy results in the reversal of fibrosis/cirrhosis and continued histological improvement in patients with chronic hepatitis B. Hepatology.

[13] World Health Organization. Guidelines on hepatitis B and C testing. Geneva: World Health Organization; 2017. Available: http://apps.who.int/iris/bitstream/10665/254621/1/9789241549981-eng.pdf?ua=1.

[14] Atkins D, Best D, Briss PA, Eccles M, Falck-Ytter Y, Flottorp S, Guyatt GH, Harbour RT, Haugh MC, Henry D (2004). Grading quality of evidence and strength of recommendations. BMJ.

[15] Surowiecki J (2004). The wisdom of crowds : why the many are smarter than the few and how collective wisdom shapes business, economies, societies, and nations.

[16] Zhang Y, Kim J, Liu F, Tso L, Tang W, Wei C, Bayus B, Tucker JD (2015). Creative contributory contests (CCC) to spur innovation in sexual health: two cases and a guide for implementation. Sex Transm Dis.

[17] Ong J, Bilardi J, Tucker JD (2017). Wisdom of the crowds: Evaluation of a crowdsourced logo contest for an international HIV conference. Sex Transm Dis..

[18] Liu C, Mao J, Wong T, Tang W, Tso LS, Tang S, Zhang Y, Zhang W, Qin Y, Chen Z (2016). Comparing the effectiveness of a crowdsourced video and a social marketing video in promoting condom use among Chinese men who have sex with men: a study protocol. BMJ Open.

[19] Tang W, Han L, Best J, Zhang Y, Mollan K, Kim J, Liu F, Hudgens M, Bayus B, Terris-Prestholt F (2016). Crowdsourcing HIV testing: a pragmatic, non-inferiority randomized controlled trial in China. Clin Infect Dis.

[20] Fourth annual national HIV/AIDS youth story-writing and video contests. AIDS Patient Care STDs. 2007;21(10):784–5. 17992723

[21] Catallozzi M, Ebel SC, Chavez NR, Shearer LS, Mindel A, Rosenthal SL (2013). Understanding perceptions of genital herpes disclosure through analysis of an online video contest. Sex Transm Infect.

[22] Morton S, Tuff R, Beckwith K, Banks M, Dixon-Terry E (2005). A Children’s poster contest on healthy eating. Calif J Heal Promot.

[23] Inglés-Camats G, Presno-Rivas MM, Antonijoan M, Garcia-Panella O, Forrest T (2012). Yummy tricks: a serious game for learning healthy eating habits. Stud Health Technol Inform.

[24] Rogers EA, Fine SC, Handley MA, Davis HB, Kass J, Schillinger D (2016). Engaging minority youth in diabetes prevention efforts through a participatory, spoken-word social marketing campaign. Am J Health Promot.

[25] Dawson AL, Hamstra AA, Huff LS, Gamble RG, Howe W, Kane I, Dellavalle RP (2011). Online videos to promote sun safety: results of a contest. Dermatol Rep.

[26] Hall D, Kline M, Glanz K (2011). Analysis of participatory photojournalism in a widely disseminated skin cancer prevention program. Health Promot Pract.

[27] Croghan IT, Campbell HM, Patten CA, Croghan GA, Schroeder DR, Novotny PJ (2004). A contest to create media messages aimed at recruiting adolescents for stop smoking programs. J Sch Health.

[28] Pan S, Stein G, Bayus B, Tang W, Matthews A, Wang C, Wei C, Tucker JD. Systematic review of innovation design contests for health: spurring innovation and mass engagement. BMJ Innovations. In Press. 10.1136/bmjinnov-2017-000203 PMC5863925 29576873

[29] Davis R (2003). Kids campaign against tobacco. Tob Control.

[30] Tang W, Mao J, Liu C, Mollan K, Li H, Wong T, Zhang Y, Tang S, Hudgens M, Qin Y, Ma B, Liao M, Yang B, Ma W, Kang D, Wei C, Tucker JD, SESH study group (2016). Crowdsourcing health communication about condom use in men who have sex with men in China: a randomised controlled trial. Lancet.

[31] World Health Organization. Consolidated guidelines on HIV testing services. Geneva: World Health Organization; 2015. Available: http://apps.who.int/iris/bitstream/10665/179870/1/9789241508926_eng.pdf?ua=1&ua=1.

[32] Moss T, Martin CW, Klausner JD, Brown BJ (2014). Integration of screening for syphilis, hepatitis C, and other sexually transmitted infections with HIV testing in a community-based HIV prevention program in Miami**,** Florida. LGBT Health.

[33] Cocoros N, Nettle E, Church D, Bourassa L, Sherwin V, Cranston K, Carr R, Fukuda HD, DeMaria A (2014). Screening for hepatitis C as a prevention enhancement (SHAPE) for HIV: an integration pilot initiative in a Massachusetts County correctional facility. Public Health Rep.

[34] Tucker JD, Bien CH, Peeling RW (2013). Point-of-care testing for sexually transmitted infections: recent advances and implications for disease control. Curr Opin Infect Dis.

[35] Xia YH, Chen W, Tucker JD, Wang C, Ling L (2013). HIV and hepatitis C virus test uptake at methadone clinics in southern China: opportunities for expanding detection of bloodborne infections. BMC Public Health.

[36] Stopka TJ, Marshall C, Bluthenthal RN, Webb DS, Truax SR (2007). HCV and HIV counseling and testing integration in California: an innovative approach to increase HIV counseling and testing rates. Public Health Rep.

[37] Ford N, Wiktor S, Kaplan K, Andrieux-Meyer I, Hill A, Radhakrishnan P, Londeix P, Forette C, Momenghalibaf A, Verster A (2015). Ten priorities for expanding access to HCV treatment for people who inject drugs in low- and middle-income countries. Int J Drug Policy.

[38] Duan ZP, Jia JD, Hou JL, Lou L, Tobias H, Xu XY, Wei L (2014). Current Challenges and the Management of Chronic Hepatitis C in Mainland China. J Clin Gastroenterol..

[39] Easterbrook PJ, Group WHOGD (2016). WHO to test and how to test for chronic hepatitis C infection - 2016 WHO testing guidance for low- and middle-income countries. J Hepatol.

[40] Lemoine M, Nayagam S, Thursz M (2013). Viral hepatitis in resource-limited countries and access to antiviral therapies: current and future challenges. Future Virol.

[41] Casey MK, Allen M, Emmers-Sommer T, Sahlstein E, Degooyer D, Winters A, Wagner AE, Dun T (2003). When a celebrity contracts a disease: the example of Earvin “magic” Johnson’s announcement that he was HIV positive. J Health Commun.

[42] Beck CS, Chapman SMA, Simmons N, Tenzek KE, Ruhl SM (2015). Celebrity health narratives and public health.

[43] Seeff LB (2002). Natural history of chronic hepatitis C. Hepatology.

[44] Orkin C, Flanagan S, Wallis E, Ireland G, Dhairyawan R, Fox J, Nandwani R, O’Connell R, Lascar M, Bulman J (2016). Incorporating HIV/hepatitis B virus/hepatitis C virus combined testing into routine blood tests in nine UK emergency departments: the “going viral” campaign. HIV Med.

[45] O’Connell R (2015). #GoingViral3in1 gone viral? Assessing impact of twitter in an HIV, hepatitis B and hepatitis C testing campaign in 10 emergency departments in the UK.

[46] Coyle C, Kwakwa H (2016). Dual-routine HCV/HIV testing: Seroprevalence and linkage to Care in Four Community Health Centers in Philadelphia, Pennsylvania. Public Health Rep.

[47] Coyle C, Kwakwa H, Viner K (2016). Integrating routine HCV testing in primary care: lessons learned from five federally qualified health centers in Philadelphia, Pennsylvania, 2012-2014. Public Health Rep.

[48] Coyle C, Viner K, Hughes E, Kwakwa H, Zibbell JE, Vellozzi C, Holtzman D, Centers for Disease C, Prevention (2015). Identification and linkage to care of HCV-infected persons in five health centers - Philadelphia, Pennsylvania, 2012-2014. MMWR Morb Mortal Wkly Rep.

[49] Zuure FR, Davidovich U, Coutinho RA, Kok G, Hoebe CJ, van den Hoek A, Jansen PL, van Leeuwen-Gilbert P, Verheuvel NC, Weegink CJ (2011). Using mass media and the internet as tools to diagnose hepatitis C infections in the general population. Am J Prev Med.

