# Supplementary material for: The HepTestContest: a global innovation contest to identify approaches to hepatitis B and C testing
Source: BMC Infect Dis. 2017 Nov 1;17(Suppl 1):701. doi: 10.1186/s12879-017-2771-4 (PMC5688427; doi:10.1186/s12879-017-2771-4)
Supplement: Additional file 1: — HepTestContest Call for Entries. This provides the logo, call for entries, and related information released to solicit entries. (DOCX 38 kb) [file 12879_2017_2771_MOESM1_ESM.docx]

**Additional File 1.**


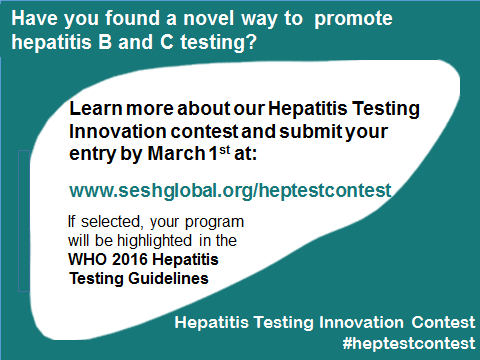


**Call for contributions**

Our SESH (Social Entrepreneurship for Sexual Health) team in collaboration with the WHO Global Hepatitis Programme is organizing an innovation contest on hepatitis B and C testing.   The purpose of this contest is to identify innovative examples of delivering hepatitis B and C testing that will be highlighted within the 2016 WHO Hepatitis Testing Guidelines.   The innovation could be delivery to a new population, in a new setting (e.g. prisons), or a new messaging approach to encourage testing uptake (e.g. social media). The goal is to identify interventions aimed to increase testing, providing real-world examples to be used alongside the WHO Hepatitis Testing Guidelines. An announcement is attached and details are below.  Your submission, in the form of a 300-500 word description of your innovation and its impact, must be submitted by March 1, 2016. Submissions in English are encouraged; however, we will accept entries in Arabic, Chinese, French, Russian, and Spanish. Send us your creative contribution!

Video introduction: https://www.youtube.com/watch?v=wAJuvnoUNjA

More information and to submit: [www.seshglobal.org/heptestcontest](http://www.seshglobal.org/heptestcontest)

Questions: [hepatitisinnovation@gmail.com](mailto:hepatitisinnovation@gmail.com) or Kathrine Meyers at [kmeyers@adarc.org](mailto:kmeyers@adarc.org)

**Further instructions**

Please submit a 300-500 word description of your innovative way of promoting hepatitis B or C testing.  The entry should include four components:

1) TESTING MODEL:  a description of how you promote hepatitis B or C testing and how testing happens;

2) INNOVATION: a description of what is novel with regard to promoting uptake of testing (eg. setting, population, messaging etc.);

3)  EFFECTIVENESS:  data to support increased uptake of testing (focused on number of tests performed, but including data on people linked to care, if available)

4) NEXT STEPS: plan for continuing this work.

The entries will be judged by a diverse panel of experts based on the four criteria listed above.  Selections will be made in March, with the top entries receiving feedback and an opportunity to resubmit a revised version to be included in the WHO Hepatitis Testing Guidelines.

**Contest Steering Committee**

Isabelle Andrieux-Meyer (Médecins Sans Frontières, Switzerland), Tasnim Azim (International Center for Diarrhoeal Disease Research Center, Bangladesh), Philippa Easterbrook (WHO, Switzerland), Carmen Figueroa (WHO, Switzerland), Charles Gore (Hepatitis C Trust and World Hepatitis Alliance, UK), Karyn Kaplan (Asia Catalyst, USA), Giten Khwairakpam (TREAT Asia/amfAR, Thailand), Kathrine Meyers (Aaron Diamond AIDS Research Center), Veronica Miller (Forum for Collaborative HIV Research, USA), Antons Mozalevskis (WHO/Europe, Denmark), Michael  Ninburg (Hepatitis Education Project, USA), Ponsiano Ocama (Makerere University, Uganda), Rosanna Peeling (London School of Hygiene and Tropical Medicine and International Diagnostics Centre, UK), Razia Pendse (WHO-SEARO, India), Gabriele Riedner (WHO-EMRO, Egypt) , Joseph Tucker (University of North Carolina, SESH, and the International Diagnostics Centre, China), Nick Walsh (WHO-WPRO)

**WHO Hepatitis resources**

General Hepatitis: <http://www.who.int/hepatitis/en/>

WHO Chronic Hepatitis B Guidelines: <http://who.int/hepatitis/publications/hepatitis-b-guidelines/en/>

WHO Chronic Hepatitis C Guidelines: <http://who.int/hepatitis/publications/hepatitis-c-guidelines/en/>
